# Convergent microevolution of *Cryptococcus neoformans* hypervirulence in the laboratory and the clinic

**DOI:** 10.1038/s41598-017-18106-2

**Published:** 2017-12-20

**Authors:** Samantha D. M. Arras, Kate L. Ormerod, Paige E. Erpf, Monica I. Espinosa, Alex C. Carpenter, Ross D. Blundell, Samantha R. Stowasser, Benjamin L. Schulz, Milos Tanurdzic, James A. Fraser

**Affiliations:** 1 Australian Infectious Diseases Research Centre, The University of Queensland, Brisbane, Queensland Australia; 2 School of Chemistry & Molecular Biosciences, The University of Queensland, Brisbane, Queensland Australia; 3 School of Biological Sciences, The University of Queensland, Brisbane, Queensland Australia

## Abstract

Reference strains are a key component of laboratory research, providing a common background allowing for comparisons across a community of researchers. However, laboratory passage of these strains has been shown to lead to reduced fitness and the attenuation of virulence in some species. In this study we show the opposite in the fungal pathogen *Cryptococcus neoformans*, with analysis of a collection of type strain H99 subcultures revealing that the most commonly used laboratory subcultures belong to a mutant lineage of the type strain that is hypervirulent. The pleiotropic mutant phenotypes in this H99L (for “Laboratory”) lineage are the result of a deletion in the gene encoding the SAGA Associated Factor Sgf29, a mutation that is also present in the widely-used H99L-derived KN99**a**/α congenic pair. At a molecular level, loss of this gene results in a reduction in histone H3K9 acetylation. Remarkably, analysis of clinical isolates identified loss of function *SGF29* mutations in *C. neoformans* strains infecting two of fourteen patients, demonstrating not only the first example of hypervirulence in clinical *C. neoformans* samples, but also parallels between *in vitro* and *in vivo* microevolution for hypervirulence in this important pathogen.

## Introduction

Reference strains are an essential element of laboratory research that provide a common genotype to enable comparison of scientific observations in any given species across a community of researchers. Whilst key reference strains are usually maintained in centralised repositories with defined storage conditions, they are also commonly passed from laboratory to laboratory where storage methods can vary from repeated subculture on agar slants at room temperature to freezing at −80 °C^1–4^. The inevitable accumulation of mutations during repeated passaging or cryopreservation can produce divergent lineages that differ from the original reference genotype, potentially undermining the *raison d*’*être* of a reference strain.

The epitome of reference strain divergence is *Escherichia coli* strain K-12. Isolated in Palo Alto, California in 1922 from the stool of a convalescent diphtheria patient^5^, *E. coli* K-12 is one of the most widely used reference strains. An analysis of K-12 stored in a stab culture for 30 years revealed the development of massive heterogeneity even in this state of very slow growth; of 118 colonies purified from this stab, 68 different insertion sequence-associated RFLP patterns were observed^6^. While genetic diversity such as this may be dismissed as a consequence of the storage method, mutations have also been described in K-12 subcultures obtained from stock centres where strains are frozen^7,8^. Thus, even very infrequent subculturing still provides ample opportunity for microevolution and the emergence of genetic heterogeneity.

Laboratory maintenance of cultures may therefore be viewed as a very slow mutation accumulation experiment, with ongoing selection taking place with each subculture. Laboratory passage often leads to reduced fitness; in the past subculturing has led to the attenuation of virulence in isolates of pathogens including *Burkholderia pseudomallei* ^9^, *Mycobacterium tuberculosis* ^10^, *Cryptococcus deneoformans* ^11^ and *Cryptococcus neoformans* ^12^. As the key trait of interest in a pathogenic species is virulence, any alteration to the ability of a reference strain to cause disease is important.

*C. neoformans* is a basidiomycete yeast that primarily infects the immunocompromised population, disseminating *via* the lungs to the central nervous system causing life-threatening meningoencephalitis^13–15^. Even with the best available treatments, mortality rates in developed countries can be up to 20%. In less privileged countries where treatment availability is limited, there is a much poorer patient prognosis, with mortality rates as high as 75%^16,17^. The impact of the disease is significant, especially in those populations with restricted access to health care, leading to an estimated 600,000 deaths per year^16,17^.

Our understanding of this pathogen has grown considerably over the last four decades, a process that has been greatly facilitated by many in the field employing common strains for their analyses. The best example of these is *C. neoformans* type strain H99 (ATCC 208821), which was originally isolated on February 14 1978 by Dr John Perfect at Duke University Medical Center from a 28-year-old male with Hodgkin’s disease^12^. Since that time, H99 has been distributed to numerous research laboratories worldwide and has become the most common reference strain used in the *C. neoformans* research community. As with all type strains, H99 exhibits some unique features that are not representative of all isolates of the species. For example, the molecular characterization of H99 has revealed the presence of a unique translocation that disrupts two genes, influencing glucose metabolism at human body temperature and melanin production, two key virulence-associated traits^18^.

**Figure 1:**
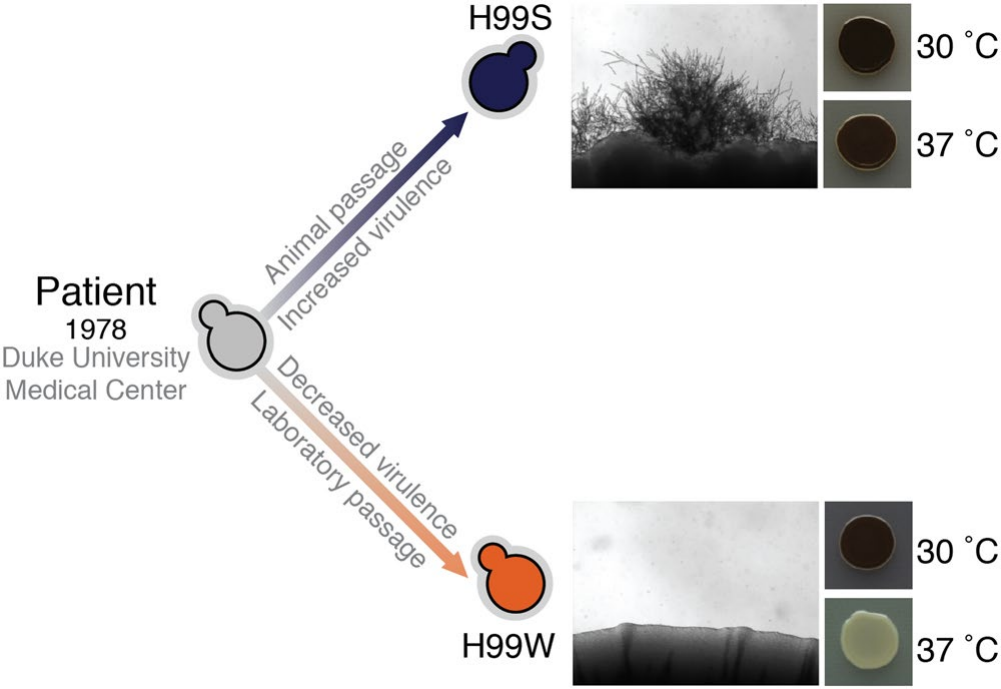
It was proposed during the H99 genome project that H99O was the genotype of the original patient isolate, and that it has diverged into two phenotypically distinct lineages since isolation. H99S (Stud) was derived by Dr John Perfect following passage through a rabbit central nervous system infection model and exhibits enhanced virulence. H99W (Wimp) arose following repeated passaging on laboratory medium and is hypovirulent. H99S exhibits prolific mating (V8 media) and high melanisation (l-DOPA media) at 37 °C, while H99W is attenuated for both phenotypes.

As with other reference strains, H99 has also been shown to have accumulated mutations during laboratory passage. During the sequencing of the H99 genome, two divergent lineages descended from an “Original” H99 strain H99O were identified (Fig. 1)^12^. H99S (for “Stud”) arose following passage through a rabbit, and exhibits enhanced melanin production at 37 °C and increased virulence. H99W (for “Wimp”) accidentally arose during the sharing of H99 between laboratories, and mates poorly, produces less melanin at 37 °C and has severely attenuated virulence. Sequencing and subsequent mutagenesis experiments revealed the H99W phenotype to be caused by a 7 bp insertion in a gene predicted to encode a glutamine-rich protein with a dimerization LisH domain, which was subsequently named *LMP1* ^12^. The H99W lineage has now been largely abandoned by the community, as has the genome deletion set it was used to create^19^.

Here, to further elucidate the laboratory history of H99, we have sequenced an additional nine subcultures obtained from some of the most prolific *C. neoformans* laboratories around the world. Analysis of these data revealed that the most commonly used laboratory subcultures actually belong to a previously uncharacterised third lineage of H99 that has acquired a pleiotropic mutation whose phenotypes include enhanced melanin production and hypervirulence. This mutation in the gene *SGF29* correlates with a loss of histone acetylation at many locations throughout the *C. neoformans* genome. Remarkably, we identified independently acquired loss of function mutations in *SGF29* in multiple clinical isolates, demonstrating parallels between *in vitro* and *in vivo* microevolution for enhanced virulence in this important pathogen.

## Results

### The original H99 from 1978 may no longer exist

Our first insights into heterogeneity in the H99 family tree arose during the genome sequencing project^12^. DNA from subculture H99O was Sanger sequenced to generate a high quality reference genome, while DNA from subcultures H99S, H99W and the H99W-related H99ED (for “Eunuch Duke”) were sequenced using Illumina technology^12^. Together, these data enabled the identification of two distinct lineages that appeared to derive from H99O, and revealed a mutation in *LMP1* (for “Low Mating Performance”) in H99W and descendent strains that severely compromised fecundity and virulence^12^.

To build on these initial observations and complete the H99 pedigree, we employed Illumina technology to sequence the genome of an additional nine H99 subcultures. Two of these were well-established strains discussed in the genome paper, H99E and H99C, both known to have been derived from the H99W lineage^12^. Originally thought to be a standard subculture, H99ED had been sent from Duke University to Washington University where it was later renamed H99E (for “Eunuch”) after being distributed to the University of California, San Francisco to become H99C (for “CM018”). The remaining seven subcultures were obtained from some of the most prolific *Cryptococcus* laboratories around the world, and represent the genetic backgrounds in which the majority of *C. neoformans* molecular genetics has been performed.

**Figure 2:**
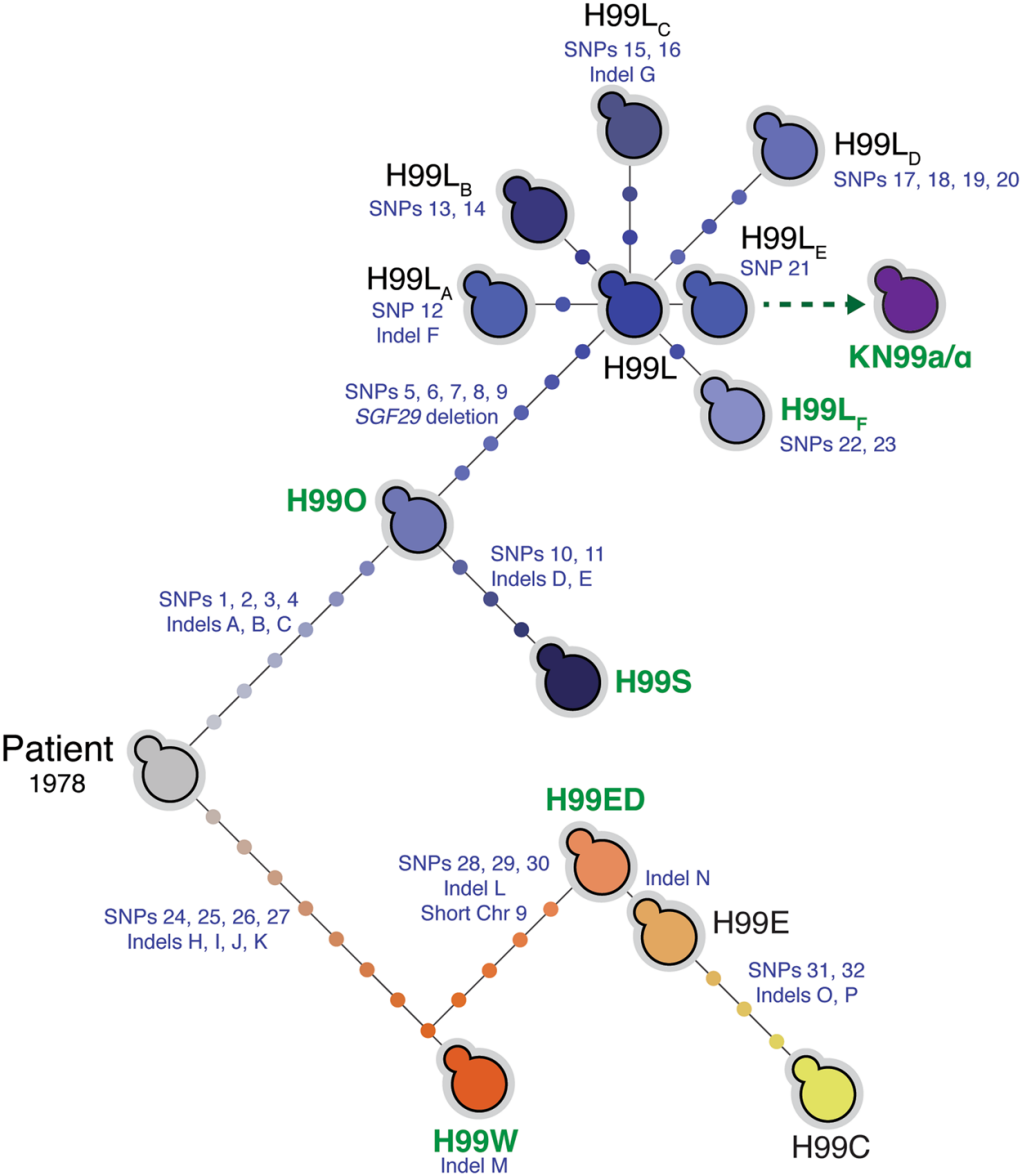
Multiple lineages have emerged from the *C. neoformans* type strain H99 since its isolation in 1978. Whole-genome sequencing data revealed that most laboratory reference strains derived from the original patient-derived H99 isolate contain unique mutations. Mutations separating each strain are denoted by a circle, the details of which are provided in Tables S4 and S5. Strains previously sequenced^12^ are in green; H99L_F_ was referred to in our previous analyses as H99F. At the request of a number of researchers that contributed strains for this analysis, the H99L strains have been anonymized.

Comparison of the nine new genome sequences, combined with reanalysis of the H99O, H99S, H99W and H99ED genome data, identified a total of 32 SNPs (Table S1), 16 small indels (Table S2) and one large 734 bp deletion across the 13 strains analysed. Each mutation was verified by Sanger sequencing. No transposon movement or gross chromosomal rearrangements were detected. Using these data, combined with sequence from the H99 congenic mating pair strain KN99α, we were able to construct an improved, comprehensive phylogeny of H99 which revealed the type strain pedigree is in fact comprised of three distinct lineages rather than two as previously thought: the H99W and H99S lineages, plus a third lineage comprised entirely of the H99 subcultures we collected from various molecular genetics laboratories which we have dubbed the “Laboratory” lineage H99L (Fig. 2). The H99L lineage includes H99L_E_, the subculture employed in the creation of the widely-used KN99**a**/α congenic pair.

The detailed H99 phylogeny enabled us to ask a fundamental question: which strain of H99 came from the patient in 1978? By comparing the identified mutations to a variety of clinical and environmental *C. neoformans* genomes, we were able to establish the likely common ancestor of the sequenced H99 subcultures as an intermediate between H99O and H99W, a genotype that is not represented by any of the available strains (Fig. 2, Tables S1 and S2). There are two alternative models that could give rise to these data. First, H99W and H99O may have arisen within the heterogeneous population of microevolving *C. neoformans* cells proliferating in the patient, and were copurified by the Duke University Medical Center Clinical Microbiology Laboratory. Second, the Patient strain may have accumulated mutations during laboratory subculture to give rise to H99W and H99O; it may have even accumulated mutations during subculture prior to the divergence of these strains, making the original genotype impossible to determine. This strain may still reside in a long-forgotten stock at Duke University Medical Center, with a group whose subculture we did not analyse, or may simply have been lost sometime in the last four decades.

### The majority of *C. neoformans* molecular genetic studies have been performed in a compromised pleiotropic mutant H99 lineage

While the precise circumstances surrounding the origin of H99O and H99W are unknown, the ancestry of the new, discrete Laboratory lineage in the pedigree can be more easily explained. The acquisition of enhanced virulence in H99S occurred following passage through a rabbit. As this enhanced virulence characteristic is not shared with H99O, the progenitor of the H99S and Laboratory lineages, the most parsimonious explanation is that the Laboratory strains instead arose during separate, routine passage of H99O. The Laboratory lineage is represented by a cluster of all seven of the subcultures we collected from various molecular genetics laboratories around the world. All share the same five SNPs and one deletion; one strain bears only these shared mutations, and we have therefore named this prototypical strain H99L (for “Laboratory”; Fig. 2). The other six strains differ from H99L by one to four unique mutations, and we have named these strains H99L_A–F_; subculture H99L_F_ was previously referred to as H99F^12^ (Fig. 2, Tables S1 and S2).

**Figure 3:**
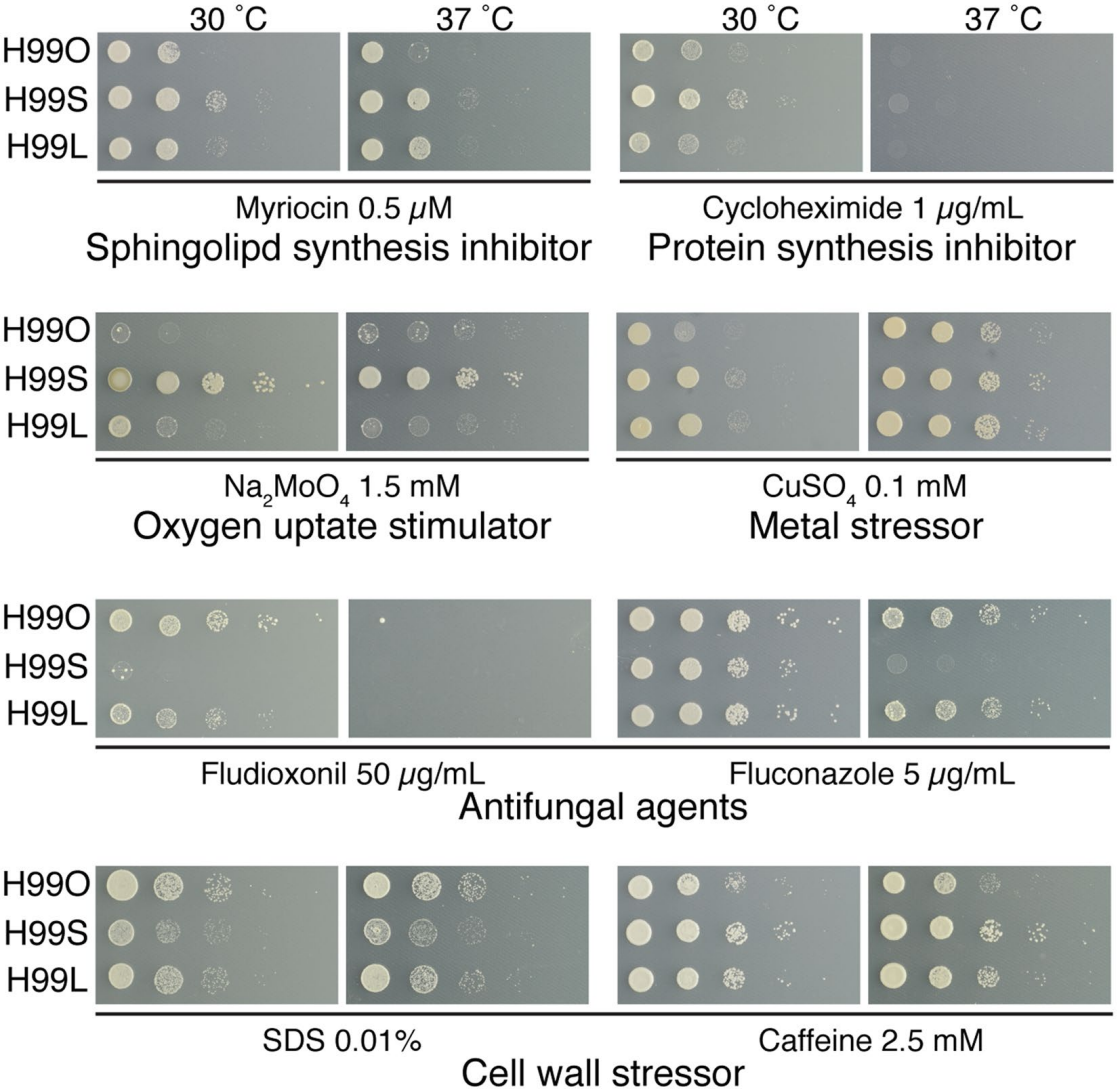
Phenotypic variation exists between widely used *C. neoformans* reference strains. Despite their close relationship, multiple phenotypic differences exist between key H99 subcultures. Spotting assays were performed with serial 10-fold dilution of cells on YNB media supplemented with 2% glucose and 10 mM ammonium sulfate, with test compounds added at the concentrations indicated.

The finding that H99S and H99L represented such distinct lineages was surprising; the Laboratory strains have repeatedly been shown to exhibit virulence and melanin production equivalent to the hypervirulent H99S, and hence were previously hypothesised to be closely related^12,20–26^. However, closer analysis with a range of *in vitro* growth assays revealed that while the lineages often exhibit equivalent phenotypes, in some instances they are substantially different (Fig. 3). The number of differences seen from just a few *in vitro* phenotypic tests supports the model that these strains have each acquired pleiotropic mutations that have far-reaching implications in cell physiology. We therefore hypothesised that one of the mutations that differentiate the H99 Laboratory lineage from H99O would be responsible for the previously observed hypervirulence and enhanced melanisation of H99L_F_^12^.

### The H99L mutant phenotype is not due to a single nucleotide polymorphism

The members of the H99L lineage all share five SNPs that differentiate them from H99O. SNP 5 maps to the large repeat-riddled centromere of chromosome 2 and SNP 6 occurs in the middle of the 301 bp 3′ UTR of gene *CNAG_00843*, predicted to encode salicylate hydroxylase. SNP 7 is 21 bp into the 54 bp third intron of a gene predicted to encode dihydroxyacetone kinase (*CNAG_02799*); this gene has a miscRNA (*CNAG_12267*) transcribed on its noncoding strand, and the SNP is 21 bp into the 78 bp fifth intron of this transcript. SNP 8 is in the 610 bp intergenic region between two convergently transcribed genes (*CNAG_00976* and *CNAG_00975*). All of these mutations are predicted to be silent.

In contrast, SNP 9 occurs in the third nucleotide of intron 3 of *CNAG_05325*. While this ORF was given no functional annotation during the genome project, it shares 81% protein identity with the glycosyl hydrolase family 88 domain predicted to be encoded by *CNAG_05991*. We therefore named the SNP 9-containing gene *GHH1* for Glycosyl Hydrolase Homologue. We observed that the SNP in this gene alters the 5′ donor site of the third intron from GCGAGT to GCCAGT, and may therefore interfere with correct mRNA maturation. Failure to splice intron 3 reliably would truncate the 219 amino acid protein just after residue 56, removing the majority of the region that shows homology to the glycosyl hydrolase family 88 domain. To investigate any potential association of *GHH1* with the enhanced melanisation and virulence of H99L, we employed biolistic transformation to delete the gene in H99O. The *ghh1∆* strain was then complemented by introducing the wild-type allele at the neutral Safe Haven site on chromosome 1^27^ to create strain *ghh1∆* + *GHH1* ^H99O^, and with the H99L mutant allele to give *ghh1∆* + *GHH1* ^H99L^. *In vitro* characterisation of the mutant and its complemented derivatives showed no difference in melanin production compared to wild-type when grown on l-DOPA media at 30 or 37 °C, indicating loss of Ghh1 function is not responsible for this phenotype in the Laboratory lineage (Supplementary Figure 1A). Furthermore, *in vivo* characterisation of *ghh1∆* and *ghh1∆* + *GHH1* ^H99O^ using a murine inhalation model of cryptococcosis established that deletion of *GHH1* was likely associated with a slight reduction in virulence (Supplementary Figure 1B), in contrast to the enhanced virulence observed in the Laboratory lineage. Fungal organ burden was unaffected by the gene deletion (Supplementary Figure 1C).

### The H99L mutant phenotype is caused by a deletion in *SGF29*

In addition to the five SNPs shared by the strains of the Laboratory cluster, we also identified a 734 bp deletion in *CNAG_06392* on chromosome 13 that arose in this lineage during its divergence from H99O. Reciprocal BLASTp analyses with *S. cerevisiae* identified the predicted product of this gene to exhibit 47% similarity to *S. cerevisiae* Sgf29, a component of the SAGA (Spt–Ada–Gcn5 acetyltransferase) histone acetylation complex^28^. Bioinformatic analysis of the *C. neoforma*ns protein also revealed the presence of two Tudor domains as in *S. cerevisiae* Sgf29^28^. *CNAG_06392* was therefore named *SGF29*.

**Figure 4:**
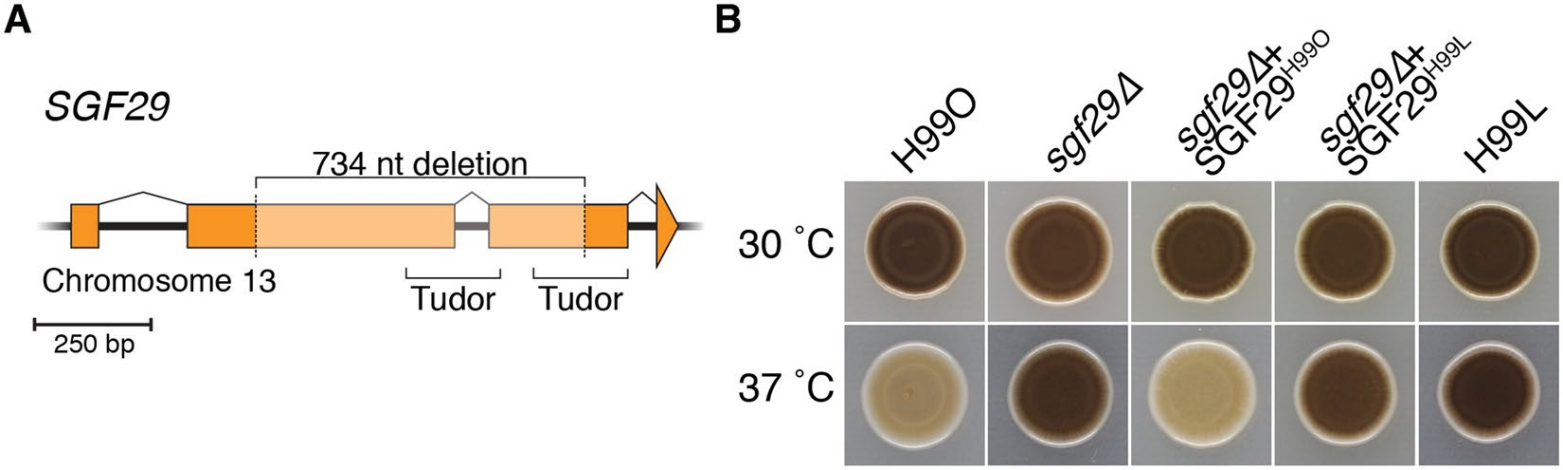
Deletion of *SGF29* results in increased melanisation at 37 °C. (**A**) The 734 bp deletion identified in the H99 Laboratory strain cluster removes the majority of the Sgf29 tandem Tudor domains. (**B**) Melanisation assayed on l-DOPA media incubated at 30 and 37 °C.

Analysis of the mutant *SGF29* allele revealed that while the 734 bp deletion in the Laboratory strains maintains the open reading frame encoding the predicted protein, it reduces the length of the product from 319 to 95 residues, removing the first Tudor domain and the majority (49 of 69 aa) of the second (Fig. 4A). As with the H99L lineage SNPs, this deletion is present in all H99L strains as well as in the KN99**a**/α congenic pair (GEO accession number PRJNA353284); the presence of the *sgf29∆* mutation in the KN99 congenic pair was also independently verified in the original glycerol stocks of these isolates (K. Nielsen, personal communication). Beyond sequencing, the presence of the *sgf29* mutation is also easily detected by a simple PCR assay as illustrated for multiple strains from the newer KN99α deletion library (“2015 Madhani Plates” and “2016 Madhani Plates”) available from the Fungal Genetics Stock Centre (Supplementary Figure 2).

To investigate whether this *SGF29* mutation was the cause of enhanced melanisation of H99L, we employed biolistic transformation of H99O to generate deletion strain *sgf29∆*. The deletion mutant was then transformed with either wild-type *SGF29* (strain *sgf29∆* + *SGF29* ^H99O^) or the mutant Laboratory allele (strain *sgf29∆* + *SGF29* ^H99L^) at the Safe Haven site on chromosome 1, with wild-type expression levels of *SGF29* verified using qRT-PCR (Supplementary Figure 3).

**Figure 5:**
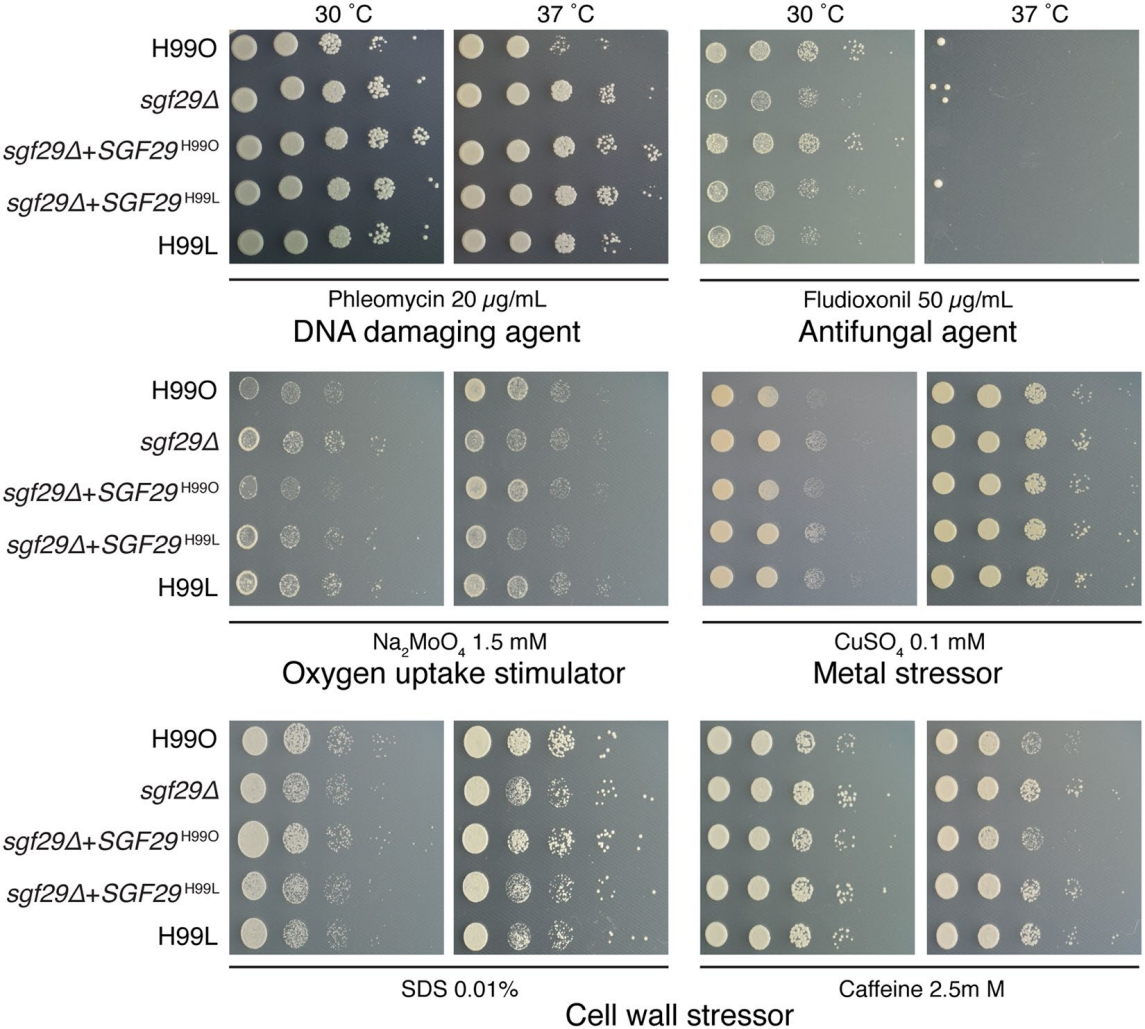
Stress assays reveal that *sgf29∆* exhibit multiple *in vitro* phenotypes. 10-fold serial dilutions of indicated strains were spotted onto YNB 2% glucose 10 mM ammonium sulfate media containing various test compounds added at the concentrations indicated and incubated for 3 days at 30 and 37 °C.

Subsequent testing of melanin production on l-DOPA medium revealed that while these strains were indistinguishable from H99O at 30 °C, deletion of *SGF29* resulted in enhanced melanisation at 37 °C, consistent with the phenotype of strains from the Laboratory lineage (Fig. 4B). This result fully supported our hypothesis that the H99L lineage had acquired a mutation since divergence from H99O that enhanced melanisation *in vitro*. Furthermore, transformation of the H99O *sgf29∆* strain with the wild-type allele was sufficient to complement this defect, while the strain bearing the H99L deletion allele remained hypermelanised. Additional subtle but reproducible *in vitro* phenotypic differences observed between the Laboratory lineage strains and H99O could also be correlated with the absence of *SGF29*. Like strains of the Laboratory lineage, the H99O *sgf29∆* mutant displayed increased resistance to caffeine, myriocin and sodium molybdate, and decreased resistance to sodium dodecyl sulfate and fludioxonil. Intriguingly, while some of the observed mutant phenotypes were complemented following reintroduction with the wild-type allele of *SGF29*, this was not always the case. For example, the increased growth of the *sgf29∆* mutant on phleomycin was not complemented, indicating that reintroduction of functional Sgf29 is insufficient to fully address the consequences of gene deletion (Fig. 5). These results are consistent with our recent observation that lost epigenetic memory can be difficult (or even impossible) to restore in *C. neoformans* ^29^.

### The loss of *SGF29* causes hypervirulence

Arguably the most important difference between H99O and H99L is the hypervirulence of the strains in the Laboratory lineage. In the widely employed inhalation model of *C. neoformans* virulence, most infected mice succumb to infection by Laboratory lineage strains after around 20 days. In contrast, mice infected with H99O can generally survive up to around day 30^12^.

**Figure 6:**
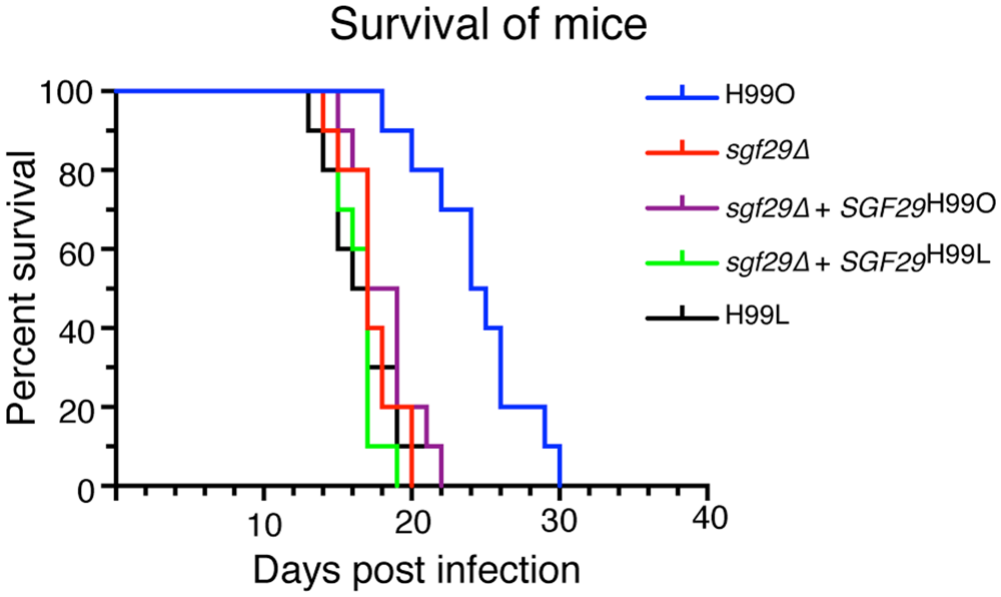
The loss of *SGF29* causes hypervirulence. Survival of mice using an inhalation model of cryptococcosis is displayed using Kaplan-Meier survival curves. Pairwise comparisons conducted using Mantel-Cox log-rank test *sgf29Δ* is hypervirulent when compared with parental strain H99O (*p* = < 0.01).

To determine whether the *sgf29* mutation in the Laboratory lineage was responsible for the observed hypervirulence phenotype, we infected mice with H99O, the H99O *sgf29∆* mutant, the complemented *sgf29∆* + *SGF29* ^H99O^ strain, a complemented *sgf29∆* + *SGF29* ^H99L^ strain and H99L. The H99O *sgf29∆* mutant displayed hypervirulence that was statistically equivalent to the virulence of H99L (*p* = 0.713; Fig. 6), consistent with the *sgf29* mutation causing the altered phenotypes of the Laboratory lineage strains. Again, this result fully supported our hypothesis that the H99L lineage had acquired a mutation since divergence from H99O that caused hypervirulence. Intriguingly, reintroduction of *SGF29* or the H99L *sgf29∆* allele into the mutant was not sufficient to abolish hypervirulence in the murine model. As we had already shown that not all of the *sgf29∆* mutant phenotypes were complemented *in vitro*, it was not entirely unexpected that the complex, multigenic trait of virulence would not be restored to wild-type, providing further valuable evidence that lost epigenetic memory in *C. neoformans* is not easily restored.

### ChIP-seq analyses reveal that histone H3 acetylation is affected by the loss of *SGF29*

Having demonstrated that *C. neoformans* Sgf29 influences a variety of *in vitro* phenotypes as well as virulence in a murine model, we proceeded to investigate its biochemical function. As the SAGA complex has been shown to play a key role in histone acetylation in a variety of species, we hypothesised that loss of *SGF29* would evoke a change in histone acetylation patterns. To test our hypothesis, we used anti-histone H3 Lysine 9 acetyl (H3K9ac) antibodies in chromatin immunoprecipitation sequencing (ChIP-seq) to isolate DNA directly associated with H3K9ac in H99O, the H99O *sgf29Δ* mutant, and H99L. Our ChIP-seq analyses identified an average of 3,200 H3K9ac regions in H99O. Many of these regions lost their H3K9ac mark in the H99O *sgf29Δ* mutant, bringing the number of H3K9ac enriched regions down to 2,600. These data provide evidence that Sgf29 influences the process of histone acetylation within the cell. Furthermore, as with wild-type, the majority of peaks identified were within 500 bp of an annotated transcription start site, indicating that loss of Sgf29 did not result in dysregulated, stochastic histone acetylation.

**Figure 7:**
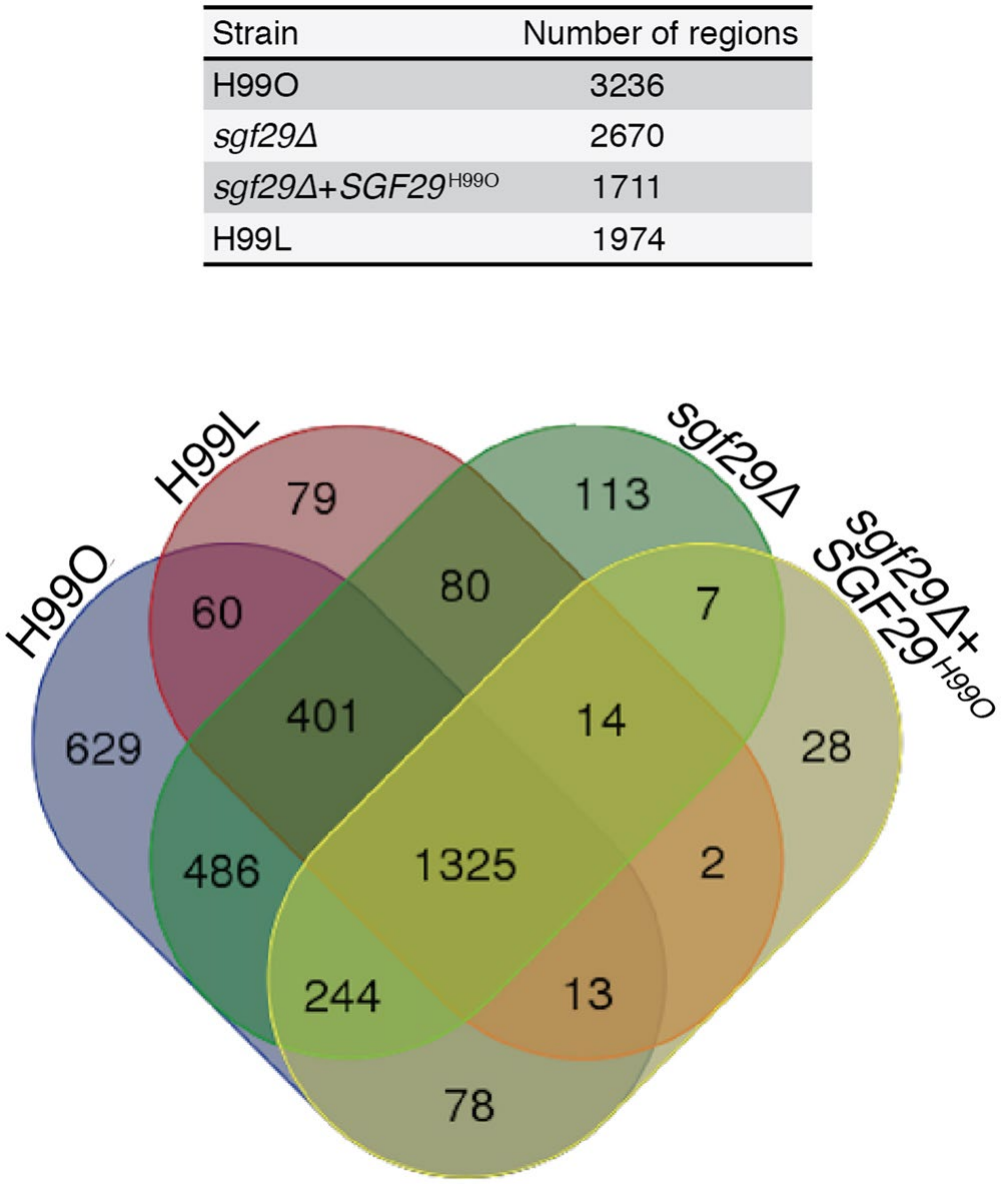
Histone H3 lysine 9 acetylation is strongly influenced by the absence of Sgf29. Venn diagram illustrating shared binding sites between the strains, 50% of ChIP-seq peaks are shared by all 3 strains, while 20% of peaks are found in H99O only.

Importantly, while there were over 700 wild-type histone acetylation regions that were absent in both the H99O *sgf29Δ* mutant and H99L, the profiles of these two strains were not identical. H99L only had around 2,000 regions, suggesting that there has been substantial epigenetic drift since the loss of Sgf29 from the Laboratory lineage (Fig. 7). To gain further insight into this observation, we also performed ChIP-seq on the complemented H99O *sgf29∆* + *SGF29* ^H99O^ strain to determine whether reintroduction of the gene could completely restore a wild-type H3K9ac pattern. Surprisingly, while reintroduction of *SGF29* restores approximately 10% of the H3K9 acetylation lost in the *sgf29∆* mutant, additional regions were absent; this strain had the lowest H3K9 acetylation of all, with only around 1,700 regions identified. This result helps explain why some phenotypes of the *sgf29∆* strain were not complemented, as reintroduction of the wild-type gene only partially restores some previously lost H3K9 acetylation, while simultaneously abolishing H3K9 acetylation elsewhere in the genome.

### Ergosterol biosynthesis illustrates the complexity of Sgf29 function

**Figure 8:**
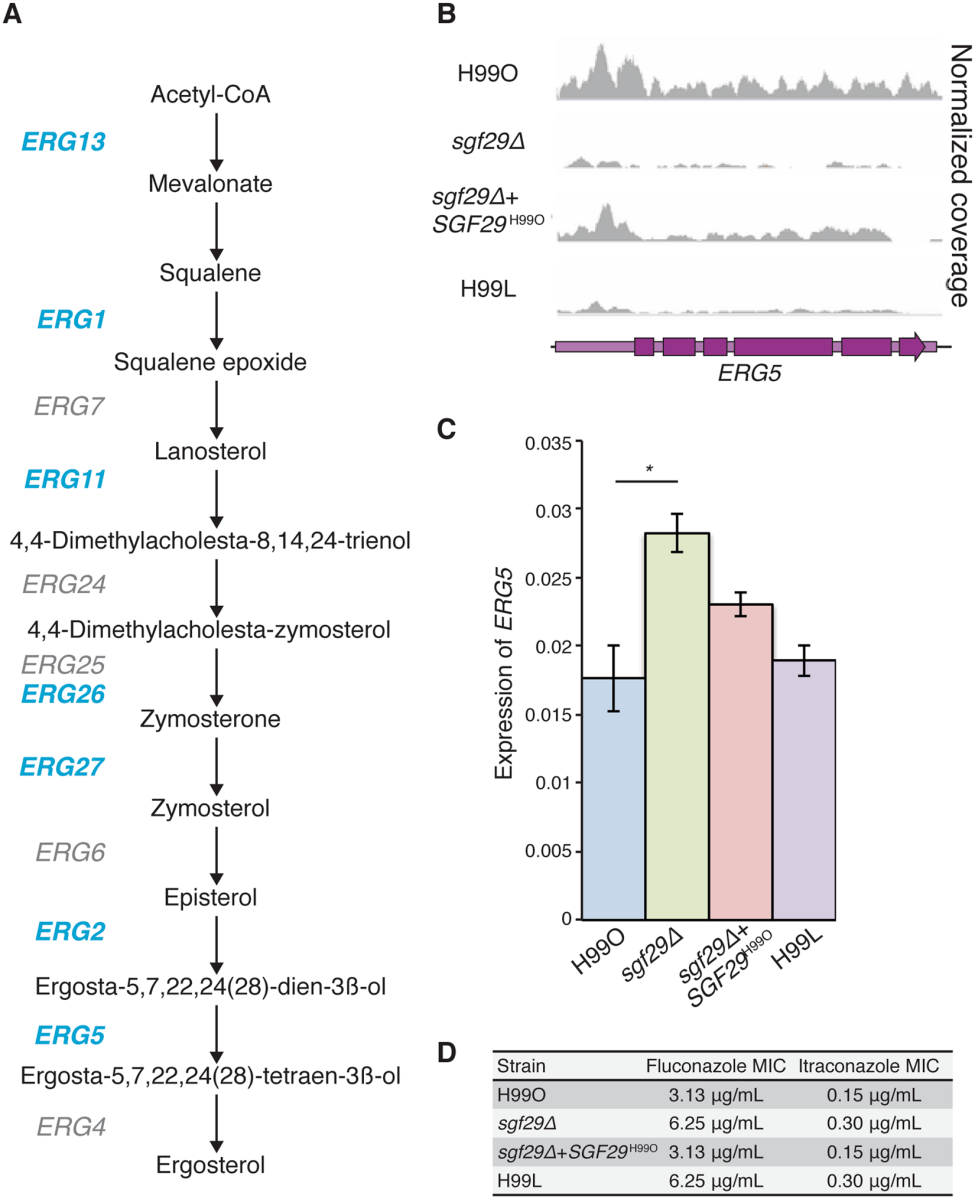
The effect of loss of *SGF29* on the ergosterol biosynthesis pathway. **(A**) Depiction of the ergosterol pathway in *C. neoformans*. Highlighted in blue are genes for which acetylation is absent in both the *sgf29∆* mutant and H99L. (**B**) ChIP-seq coverage of the *ERG5* locus. (**C**) qRT-PCR of *ERG5*. Transcript abundance is relative to *ACT1*. Values show mean, error bars show S.E.M. *p < 0.05. (**D**) MIC results show the loss of Sgf29 conferred higher resistance to the azoles fluconazole and itraconazole.

To gain insight into the direct physiological consequences of loss of functional Sgf29, we investigated several of the loci in which H3K9 acetylation had been altered. We chose to focus on ergosterol biosynthesis, as seven of the twelve genes in the pathway lost H3K9 acetylation in both the *sgf29∆* strain and H99L (Fig. 8A and B). However, of the seven genes identified, only *ERG5* resulted in a change of expression when analysed using qRT-PCR (Fig. 8C). In this instance, the *sgf29∆* mutation resulted in an increase in expression, a transcriptional response similar to that seen for regulated genes in an *S. cerevisiae sgf29∆* mutant^30^. Furthermore, no change in *ERG5* expression was observed in H99L despite the absence of a H3K9 acetylation peak, and only partial complementation was observed in *sgf29∆* + *SGF29* ^H99O^ despite the restoration of acetylation (Fig. 8C). This observation was further confounded by our MIC assays that showed an increase in resistance to fluconazole and itraconazole not only by the *sgf29∆* mutant, but H99L as well (Fig. 8D). Together, these data highlight the complexities associated with predicting the consequences of loss of *SGF29* due to the expected highly pleiotropic and often indirect effects of loss of an epigenetic regulator, particularly in the context of a multigenic phenotype. In addition to loss of H3K9 acetylation and the effects of epigenetic drift, the large number of regions influenced would have both direct and indirect effects on many biological processes in the cell.

### Convergent microevolution of hypervirulence in the laboratory and the human host

Given that loss of *SGF29* results in hypervirulence, we next investigated if mutations equivalent to that observed in H99L occur in the clinical setting. To this end, we isolated genomic DNA from a collection of 36 serial clinical isolates originating from fourteen patients in the USA and India^31^. These serial isolates were sourced from patients who had undergone relapse up to three times, providing an invaluable resource with which to investigate the genomic effects of passaging *C. neoformans* through a human host over months to years, in much the same way as our H99 pedigree followed the passage of *C. neoformans* in the laboratory.

**Figure 9:**
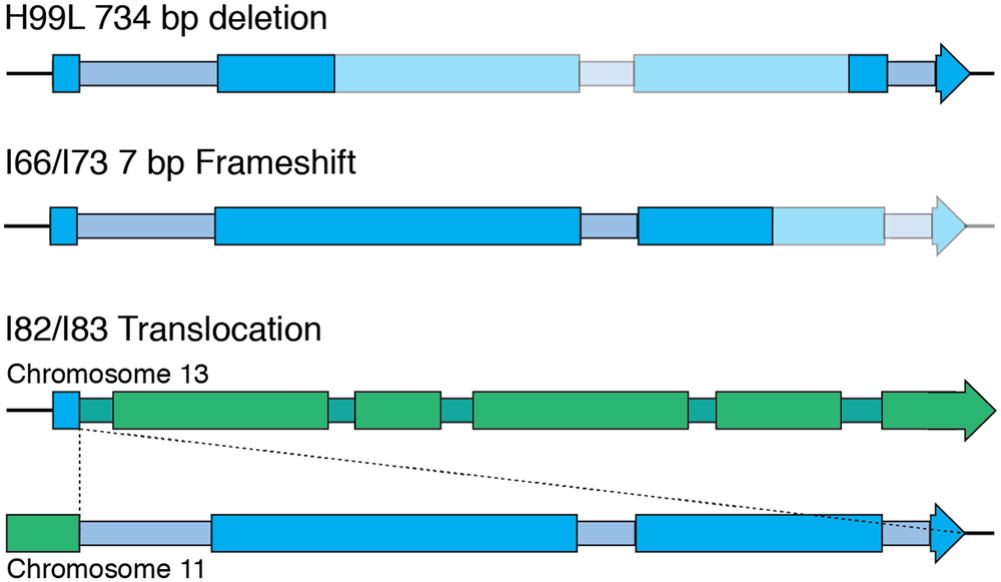
Mutations occurring in *SGF29* within H99 subtypes and serial isolates. In H99L, a 734 bp deletion has been introduced that removes amino acid residues 72 to 295 of 318, creating a new protein of 94 residues. An indel occurring in the serial isolate set I66/I73 introduces a premature stop codon at residue 274. In the serial isolate set I82/I83, a translocation occurred due to CAG microhomology within the first intron of *SGF29* and *CNAG_01704* of chromosome 11, encoding a serine/threonine protein kinase.

Remarkably, sequencing of the *SGF29* locus from the serial clinical isolate collection identified loss of function mutations in this gene in two patient series. The first series, isolates I66 and I73, were collected 131 days apart from the cerebrospinal fluid of a HIV negative patient presenting with altered sensorium in New Delhi^31^. In these strains, *SGF29* is disrupted by a frameshift within the third exon caused by a seven nucleotide deletion introducing a premature stop codon at residue 274 of 318 (Fig. 9). The second series, clinical isolates I82 and I83, were also obtained in New Delhi and were collected one day apart from the blood of a HIV negative patient presenting with fever. In these strains, there is a reciprocal translocation with CTG microhomology between the first intron of *SGF29* and the third exon of a hypothetical serine/threonine kinase gene located on chromosome 11 (*CNAG_01704*) (Fig. 9). In short, isolates from two out of fourteen patients had independent loss of function *SGF29* mutations, providing the first evidence in this pathogen of convergent microevolution of virulence in the laboratory and the clinic, as well as the presence in the clinical setting of a mutation associated with hypervirulence.

## Discussion

The *C. neoformans* type strain H99 is the most widely used reference strain within the *Cryptococcus* research community, and has been the strain employed most extensively in identifying the molecular determinants of virulence in this species. However, not all H99 strains are equivalent. We recently described the identification of a series of passage-acquired mutations that defined two lineages in this type strain^12^. Importantly, one of these mutations was an insertion in *LMP1*, whose interruption is responsible for the compromised mating and virulence associated with the H99W lineage^12^. These findings highlighted the importance of having a deep understanding of the type strains in use. In this instance, discovery of *LMP1* lead to the abandonment of this subculture, and the deletion library it was used to construct, by the community.

Here we probed deeper into the H99 family history, sequencing a further nine subcultures to gain insight into the origin of H99, and how microevolutionary events have shaped this important strain since its isolation at Duke University Medical Center in 1978. We were able to identify 32 SNPs, 16 small indels and one large 734 bp deletion that differentiated these strains and reveal that the true “Original” H99 is not H99O as previously thought, but rather a genotype that is not represented by any of the available strains and which may possibly no longer exist.

The most significant discovery to come from our study is the identification of a third, previously unknown lineage that represents the H99 subcultures used by the most prolific molecular genetic laboratories that work on this organism, and which was also used in the construction of the KN99**a**/α congenic pair. Dubbed the Laboratory lineage, these strains display melanin overproduction and hypervirulence equivalent to H99S, despite not having been passaged through an animal. Like H99S, the progenitor of this lineage is H99O, the strain for which a high quality, complete genome sequence has been determined. The strains of the H99L lineage all share 5 SNPs and one 734 bp deletion. While one of the Laboratory strains (dubbed H99L) has only these, the other six strains have acquired additional unique mutations during laboratory passage. The importance of the discovery of the Laboratory lineage lies in the effect of the mutations that define these strains. Specifically, the 734 bp deletion shared by all strains in this group removes the majority of the gene *SGF29* that encodes a component of the SAGA complex; this mutation is also present in the KN99 congenic pair, which was constructed using the subculture we are now calling H99L_E_. The work we have performed here shows this mutation causes melanin overproduction at 37 °C as well as hypervirulence, in addition to a range of other phenotypes, and that the majority of the molecular genetic work done investigating virulence in this species has been performed in a mutant background that is not representative of the species as a whole.

The development of hypervirulence following laboratory passage of H99 was unexpected. Experimental evolution in the form of mutation accumulation often leads to more generalized diminished fitness due to the collection of mutations which may be neutral in laboratory conditions but deleterious in complex physiological environments^32,33^. Experimental evolution studies demonstrate that adaptation to a particular environment can result in decreased fitness in an alternative environment^34–36^. Thus laboratory strains of pathogens, having been removed from the human host, may be losing the very traits that are under investigation. This was not the case with loss of *SGF29*.

Sgf29 was first identified as a component of the SAGA complex in 2002, and is conserved across the eukaryotes^37,38^. Similar to other “histone reader proteins” that can recognize sites for post-translational histone modifications, Sgf29 bears characteristic Tudor domains and facilitates the recruitment of specific protein complexes at the site of histone modifications^39^. In the simplest model, these Tudor domains interact with K4-methylated histone H3 to recruit SAGA, enabling acetylation of target H3 lysine residues by Gcn5, and thereby control of gene transcription through remodelling of chromatin structure^28,30,40–42^. However, the true function of Sgf29 is more complex; in *S. cerevisiae* the Sgf29-containing histone acetyltransferase HAT module (which also includes Gcn5, Ada2 and Ada3) of the SAGA complex is also part the related regulatory complexes SLIK, ADA and HAT-A2^43^. Furthermore, beyond its role in HAT, Sgf29 has been shown to perform additional roles independent of Gcn5^44^.

Two other components of the SAGA HAT module have previously been well characterised in *C. neoformans* ^20,45^. The histone acetyltransferase Gcn5 and the transcription coactivator Ada2 have both been shown to be required for successful infection of mice when studied in the H99L background. The role that loss of Sgf29 plays in this phenotype is unknown. As was expected, loss of Sgf29 lead to a reduction in histone H3K9 acetylation across the genome, with a 20% reduction in number of regions observed in the H99O *sgf29Δ* mutant. The reduction in H3K9 acetylation supported the previous analysis of H3K9 acetylation in studies of Ada2, where “wild-type” had substantially fewer regions than H99O, consistent with this study being performed in a H99L background that lacks Sgf29^45^. Furthermore, loss of Ada2 subsequently reduced H3K9 acetylation even further to around only 364 regions, highlighting that, as in *S. cerevisiae*, the effect of loss of Sgf29 is not identical to loss of other elements of the HAT module. At this point we are unable to predict what the H3K9 acetylation pattern would be in a *C. neoformans ada2∆* mutant if Sgf29 were still present, or which of the Sgf29-dependent sites are also dependent on Ada2.

Intriguingly, and perhaps one of the most fascinating features of *SGF29* in *C. neoformans*, is the inability of the wild-type gene to restore wild-type H3K9 acetylation patterns in the mutant strain upon re-introduction. The introduced construct is transcribed at wild-type levels and functional, as several *in vitro* mutant phenotypes were complemented, as was the H3K9 acetylation at a range of locations in the genome. However, most regions were not restored to their previous state, and additional H3K9 acetylation was also lost. As a consequence, the hypervirulent *sgf29∆* phenotype remained. There are strong parallels between this inability to restore lost epigenetic memory in *C. neoformans* with our recent studies investigating the sirtuins of this species; as with the *sgf29∆* mutation, sirtuin mutants in which the wild-type gene is reintroduced also do not have their epigenetic “amnesia” restored^29^.

While discovering that the majority of published work in *C. neoformans* has been performed in a hypervirulent pleiotropic mutant background, our studies also surprisingly provided clinical legitimacy for the *sgf29∆* background as a useful *C. neoformans* model. Of the strains studied from fourteen patients, two series represented strains in which independent loss of function mutations of *SGF29* had been acquired. In these instances, both isolates from the two patients contained *sgf29* mutations. We are therefore unable to determine whether the patients were infected with a hypervirulent environmental strain lacking *SGF29*, or if the loss of *SGF29* occurred early in infection as part of the microevolutionary burst this species is known to undertake during passage through the human host. Irrespective of this, the studies performed in H99L therefore reflect the infections in this smaller cohort of patients, rather than *C. neoformans* infections as a whole.

Identification of the loss of *SGF29* in the genetic background upon which the vast majority of our molecular understanding is based has substantial implications for the *Cryptococcus* research community. What is the appropriate subculture of H99 in which to study *C. neoformans*, particularly when considering the generation of a whole genome deletion library? Our data strongly indicate that the only subculture of the type strain that appropriately reflects wild-type *C. neoformans* is H99O, and that if this is not used, published works should clearly state that the studies have been performed in the hypervirulent H99L *sgf29* mutant. Furthermore, these findings provide a strong argument for the need for publications to include a statement regarding strain storage conditions, including reference to regularity of revival from frozen stocks, for the purposes of promoting consistency between laboratories. Perhaps more importantly, this work suggests that inactivation–even temporarily–of Sgf29 could be a highly effective mechanism for *C. neoformans* to rapidly undergo heritable microevolutionary change without altering its genetic material, and enhance the success of infection.

## Methods and Materials

### Strains and growth conditions

Strains used in this study are detailed in Supplementary Table 3. Strains were cultured in YPD (1% yeast extract, 2% bacto-peptone, 2% glucose) unless stated otherwise. *E. coli* Mach1 cells (Invitrogen, USA) served as the host strain for transformation and propagation of all plasmids using lysogeny broth supplemented with 100 µg/mL ampicillin (Sigma, USA)^46^. Plasmids created in this study are listed in Supplementary Table 4.

### Sequencing and genomic analysis

Strains were sequenced to approximately 50 fold coverage at BGI using 90 bp paired-end reads. Reads were trimmed using Trimmomatic 0.30^47^. Mapping to H99 reference genome was performed using BWA 0.7.5^48^ using default settings. Duplicate reads were marked using Picard 1.98 (http://www.picard.sourceforge.net) and base quality score recalibration and realignment around indels was performed using GATK 3.0^49^. SNPs and indels were identified using GATK 3.0 following best practice guidelines^50^. Larger variation was detected using Breakdancer 1.1.2^51^ and CREST^52^. Mappings were visualised and SNP associated amino acid changes were determined using CLC Genomics Workbench (Qiagen, Netherlands). Unmapped reads were collected using SAMtools^53^ and regions of zero coverage were identified using BEDTools^54^. SNPs and indels were confirmed using Sanger sequencing performed at the Australian Genome Research Facility. Primers used in this study are contained in Supplementary Table 5. Genome data is available at GEO accession number PRJNA353284.

### Phenotypic stress assays

Phenotypic plate assays were performed on YNB (without amino acids and ammonium sulfate) media supplemented with 2% glucose and 10 mM ammonium sulfate unless stated otherwise. For UV sensitivity, cells were spotted then exposed for 6 sec to UV light at 48 mJ/cm^2^ in a UV Stratalinker (Stratagene, USA). For all other assays, the stressor was added to the media immediately prior to pouring at the following concentrations: 2.5 mM caffeine (Sigma, USA), 1 μg/mL cyclohexamide (Sigma, USA), 0.1 mM CuSO_4_ (Sigma, USA), 0.5 µM Myriocin (Sigma, USA), 1.5 mM Na_2_MoO_4_ (Sigma, USA), 5 μg/mL fluconazole (Sigma, USA), 50 μg/mL fludioxonil (Sigma, USA) and 0.01% SDS (Sigma, USA). Melanization assays were performed on solid L-DOPA media supplemented with 10 mM asparagine^55^. Tenfold serial dilutions were prepared immediately prior to testing, with plates being imaged at 24–96 hr. All assays were performed at both 30 and 37 °C.

### Construction and complementation of *C. neoformans* mutant strains

The gene deletion of *SGF29* and *GHH1* from the type strain H99O were generated using PCR overlap and biolistic transformation as previously described^56^. In brief, the coding sequence was replaced with the neomycin selectable marker *NEO* using a construct created by overlap PCR combining a 1 kb fragment upstream from the start codon, the *NEO* marker and a 1 kb fragment downstream from the stop codon. Strain H99O genomic DNA and the plasmid pJAF1 were used as PCR templates^12,57^. Transformations were carried out *via* biolistic particle delivery onto media containing 100 μg/mL G418 (Sigma, USA). Stable transformants were confirmed to be correct *via* Southern blot. All mutant strains were created at least twice, from independent transformations.

To complement the mutant strains, the gene, including approximately 1 kb of the 5′ region and 1 kb of the 3′ region was amplified using Phusion High-Fidelity DNA polymerase (New England Biolabs, USA) from the *C. neoformans* type strains H99O and H99L and cloned into the nourseothricin resistance Safe Haven vector pSDMA25^27^. Plasmids were sequenced to ensure they were error free. Each plasmid was linearized using BaeI, AscI or PacI and biolistically transformed into the corresponding mutant for complementation. Stable transformants were selected on YPD supplemented with 100 μg/mL nourseothricin and were confirmed to be correct *via* Southern blot.

### Murine virulence assay

For murine infection assays, 6-week-old female BALB/c mice (Animal Resources Centre, Australia) were infected by nasal inhalation^58^. For each strain, 10 mice were inoculated with a 50 μL drop containing 5 × 10^5^ *C. neoformans* cells. A maximum of 5 mice were housed per IVC cage (Tecniplast, USA) with Bed-o’Cobs 1/8″ bedding (The Andersons, USA), Crink-l′Nest nesting material (The Andersons, USA), and cardboard as environmental enrichment. Mice were provided Rat and Mouse Cubes (Specialty Feeds, Australia) and water *ad libitum*. Each mouse was examined and weighed twice daily for the duration of the experiment, with affected mice euthanized *via* CO_2_ inhalation once body weight had decreased to 80% of pre-infection weight or they exhibited symptoms consistent with infection. Death after CO_2_ inhalation was confirmed by pedal reflex prior to dissection. The brain, lungs, liver, spleen and kidneys were collected, homogenized and plated to determine colony-forming units per gram organ weight. Kaplan-Meier survival curves were plotted using GraphPad Prism 7.0 (GraphPad Software, USA). Significance was analyzed using the log-rank test while organ burden significance was determined using a one-way ANOVA with Tukey’s multiple comparisons test. *p* values of <0.05 were considered significant.

### Ethics statement

This study was carried out in strict accordance with the recommendations in the Australian Code of Practice for the Care and Use of Animals for Scientific Purposes by the National Health and Medical Research Council. The protocol was approved by the Molecular Biosciences Animal Ethics Committee (AEC) of The University of Queensland (AEC approval no. SCMB/439/13/UQ/NHMRC). Infection was performed under methoxyflurane anaesthesia, and all efforts were made to minimize suffering through adherence to the Guidelines to Promote the Wellbeing of Animals Used for Scientific Purposes as put forward by the National Health and Medical Research Council (Australia).

### ChIP-Seq

100 mL of *C. neoformans* cells were grown to OD 0.8 in YNB (2% glucose, 10 mM ammonia sulfate) at 30 °C overnight with shaking and fixed for 20 min with formaldehyde at a final concentration of 1% (Sigma USA), followed by quenching with a final concentration of 125 mM glycine (Sigma USA). Fixed cells were collected by centrifugation and washed with TBS + 125 mM glycine, followed by a second wash with TBS only. Cells were resuspended in buffer A (50 mM HEPES pH 7.5, 140 mM NaCl, 1 mM EDTA, 1% v/v Triton X-100, 0.1% w/v sodium deoxycholate) and protease inhibitors (1 mM PMSF and 1 × protease cocktail inhibitor) and subjected to mechanical bead-beating with 0.5 mm zirconium silicate beads for 3 min at 4 °C, followed by a 1 min rest, for a total of 6 cycles. The resulting chromatin was then sheared by sonication in a Bioruptor Plus sonication device (Diagenode, USA) for 30 sec at full power output, followed by a 30 second rest, for a total of 30 cycles. The lysate was clarified *via* centrifugation and protein concentration was determined with a DC protein assay (Bio-Rad, USA). 1 mg of protein was used for immunoprecipitaion with 20 μL reserved as an input sample.

Dynabeads Protein B (Life Technologies, USA) were pre-hybridized in buffer B (50 mM HEPES pH 7.5, 500 mM NaCl, 1 mM EDTA, 1% (v/v) Triton X-100, 0.1% (w/v) sodium deoxycholate) with the Anti-acetyl-Histone H3 (Lys9) antibody (Merck, USA) for 1 hr at 4 °C with rotation on a Hula Mixer (Life Technologies, USA) prior to the addition of the protein sample, followed by incubation at 4 °C for an additional 2 hr. The protein-bound beads were washed sequentially with rotation in 1 mL buffer A, buffer B (50 mM HEPES pH 7.5, 500 mM NaCl, 1 mM EDTA, 1% (v/v) Triton X-100, 0.1% (w/v) sodium deoxycholate), buffer C (10 mM Tris-HCl pH 8.0, 250 mM LiCl, 1 mM EDTA, 0.5% (v/v) NP-40, 0.5% w/v sodium deoxycholate), and buffer D (10 mM Tris, 1 mM EDTA), with immunoprecipitated protein eluted in buffer E (50 mM Tris pH 8.0, 10 mM EDTA, 1% (w/v) SDS). Chromatin from the input sample and the IP sample was released by adding NaCl to a final concentration of 10 mM and incubation overnight at 65 °C. Samples were treated with RNase A for 30 min at 37 °C followed by pronase for 2 hr at 42 °C, and extracted using a QIAquick PCR Purification Kit (QIAGEN, Netherlands).

Purified ChIP-DNA for the input and IP samples was end repaired with Klenow DNA polymerase (New England Biolabs, USA) and purified using AMPure XP beads (Agencourt, USA). The samples were A-tailed using Klenow fragment (New England Biolabs, USA) before ligating Multiplex Oligos for Illumina (Index primer set 1) (Illumina, USA) using T4 DNA ligase (New England Biolabs, USA). Ligated indexed samples were subsequently amplified as per the kit instructions, and then gel purified to remove adapter dimers and select optimal sizes (100 to 500 bp). Libraries were 7-way multiplexed on an Illumina MiSeq flow cell using MiSeq Reagent Kit v3 (Illumina, USA).

Reads generated from the input and IP samples were aligned to the *C. neoformans* type strain H99 genome reference sequence using Bowtie. Peak calling was performed using MACS2, with a p value of <0.01 considered statistically significant. Genome data is available at GEO accession number PRJNA356476.

### Quantitative real-time PCR

*C. neoformans* strains were grown in YNB with shaking at 30 °C for 16 hours. Cultures were harvested, cell pellets frozen and lyophilized, and total RNA isolated using TRIzol reagent (Life Technologies, USA). cDNA was generated using the Superscript III First-Strand Synthesis System (Invitrogen, USA). Primers were designed to span exon–exon boundaries. Quantitative real-time PCR (qRT-PCR) was performed using SYBR Green Supermix (Applied Biosystems, USA) and an Applied Biosystems 7900HT Fast Real Time PCR System with the following cycling conditions: denaturation at 95 °C for 10 minutes, followed by amplification and quantification in 45 cycles of 95 °C for 15 seconds and 60 °C for 1 minute, with melting-curve profiling at 95 °C for 2 minutes, 60 °C for 15 seconds, and 95 °C for 15 seconds. Relative gene expression was quantified using SDS 1.3.1 (Applied Biosystems, USA) based on the 2^−ΔΔCT^ method^59^. The housekeeping actin-encoding gene *ACT1* was used as a control for normalization. One-way analysis of variance was performed using the unpaired, two-tailed *t*-test in GraphPad Prism Version 6.0c. *p*-values of <0.05 were considered statistically significant.

### Minimum inhibitory concentration (MIC) assays

The drugs fluconazole and itraconazole were diluted in water to a final concentration of 5 mg/mL and stored at −20 °C until use. Stock solutions and serial two-fold dilutions of each drug for the MIC were prepared immediately prior to testing according to the recommendations of CLSI (CLSI M27-A2) modified for *C. neoformans* ^60^. Plates were checked and scored visually on days 1, 2 and 3. MIC was defined by the lowest concentration of a specific drug that inhibits all visual growth on day 3.

## Electronic supplementary material

Supplementary material
