# Supplementary material for: Convergent microevolution of Cryptococcus neoformans hypervirulence in the laboratory and the clinic
Source: Sci Rep. 2017 Dec 20;7:17918. doi: 10.1038/s41598-017-18106-2 (PMC5738413; doi:10.1038/s41598-017-18106-2)
Supplement: Supplementary file 1 — Supplementary material [file 41598_2017_18106_MOESM1_ESM.pdf]

Convergent microevolution of *Cryptococcus neoformans* hypervirulence in the laboratory and the clinic

Samantha D. M. Arras[1,2+], Kate L. Ormerod[1,2+], Paige E. Erpf[1,2], Monica I. Espinosa[1,2], Alex C. Carpenter[1,2], Ross D. Blundell[1,2], Samantha R. Stowasser[1,2], Benjamin L. Schulz[1,2], Milos Tanurdzic[3] and James A. Fraser[1,2*]

[1]Australian Infectious Diseases Research Centre

The University of Queensland, Brisbane, Queensland, Australia

[2]School of Chemistry & Molecular Biosciences,

The University of Queensland, Brisbane, Queensland, Australia

[3]School of Biological Sciences

The University of Queensland, Brisbane, Queensland, Australia

\+ Contributed equally to the project

*Correspondence e-mail: jafraser@uq.edu.au

Supporting information

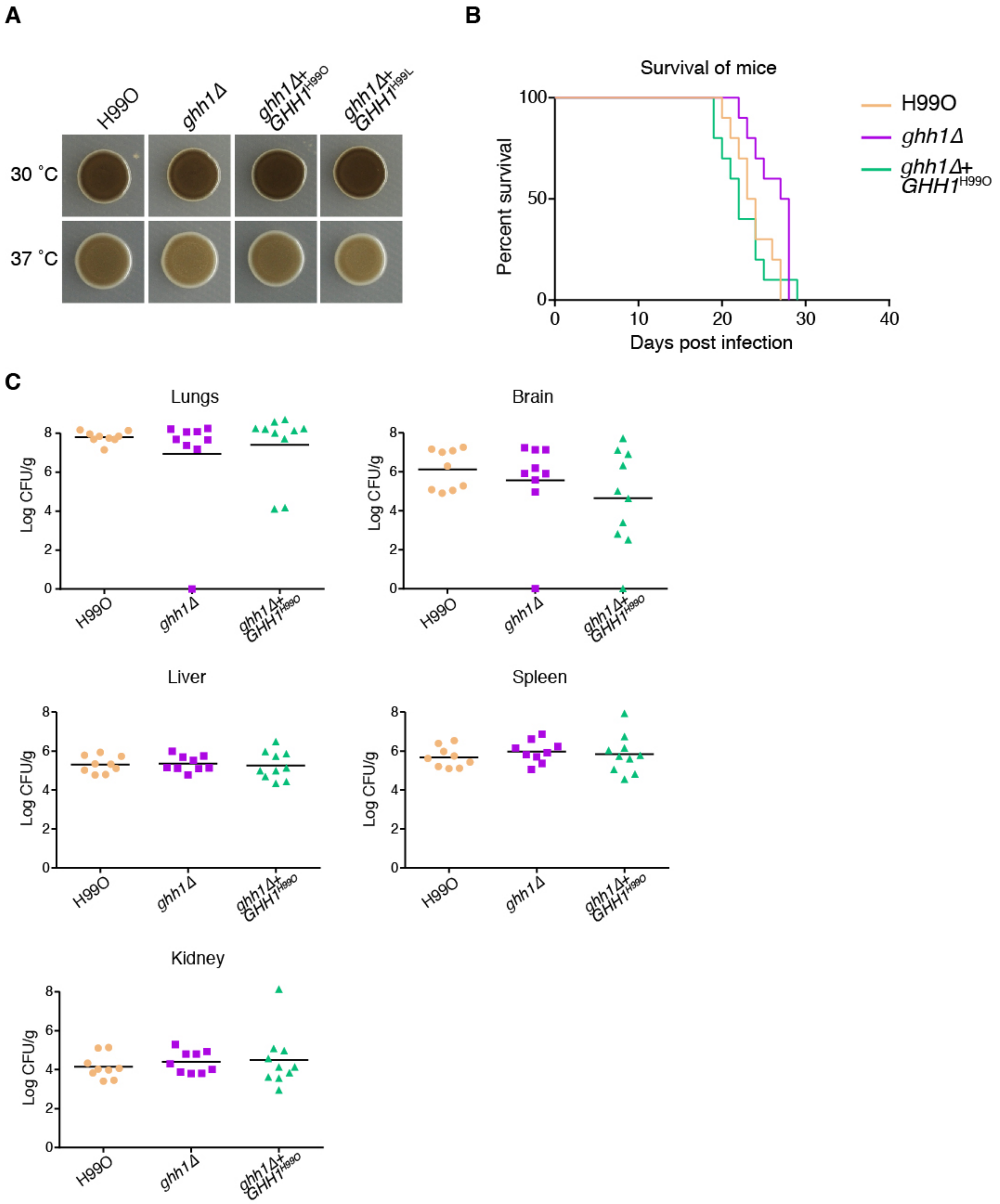

Supplementary Figure 1. Deletion of *GHH1* does not enhance virulence or melanin production in the H99O genetic background. A. Melanisation assayed on L-DOPA media incubated at 30 and 37°C. Strain *ghh1*Δ+*GHH1*^{H99O} was complemented using the wild-type H99O allele of the gene. **B.** Survival of mice using an inhalation model of cryptococcosis displayed using Kaplan-Meier

survival curves. Strain *ghh1*Δ exhibited slightly decreased virulence in comparison to H99O and to H99O and *ghh1*Δ+*GHH1*$^{\text{H99O}}$ when data for these two strains was merged (pairwise comparisons conducted using Mantel-Cox log-rank test: H99O vs *sgf29*Δ = 0.0414; H99O vs *ghh1*Δ+*GHH1*$^{\text{H99O}}$ = 1; *sgf29*Δ vs *ghh1*Δ+*GHH1*$^{\text{H99O}}$ = 0.1593 (Bonferroni adjusted P values); *sgf29*Δ vs H99O and *ghh1*Δ+*GHH1*$^{\text{H99O}}$ merge = 0.0157). **C.** Fungal burden within lungs, brain, liver, spleen and kidney was assayed at time of death using a spot dilution assay. No significant difference in fungal burden was observed between strains (one-way ANOVA global P values: lungs = 0.6144; brain = 0.2884; liver = 0.9243; spleen = 0.7086; kidney = 0.7322).

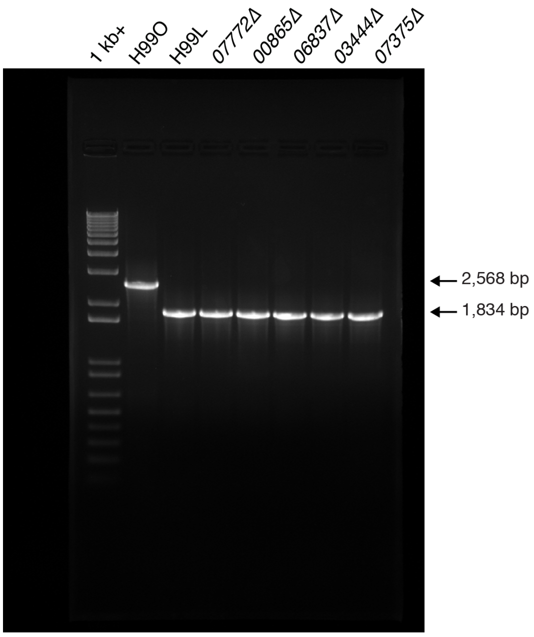

Supplementary Figure 2. The presence of the H99L *sgf29*Δ allele is easily detected with a simple PCR assay. PCR using primers UQ3574 and UQ3575 amplifies a 2568 bp *SGF29* fragment from wild-type (H99O, lane 2) and a 1834 bp band from strains bearing the H99L *sgf29*Δ allele (H99L, lane 3) easily visible on a 1% agarose TAE gel. A selection of mutants taken from the "2015 Madhani Plates" and "2016 Madhani Plates" KN99α-derived gene deletion library available from the Fungal Genetics Stock Centre (Lanes 4-8) confirms the strains in this library are constructed in a hypervirulent mutant background. Lane 1, Invitrogen 1 Kb Plus DNA Ladder.

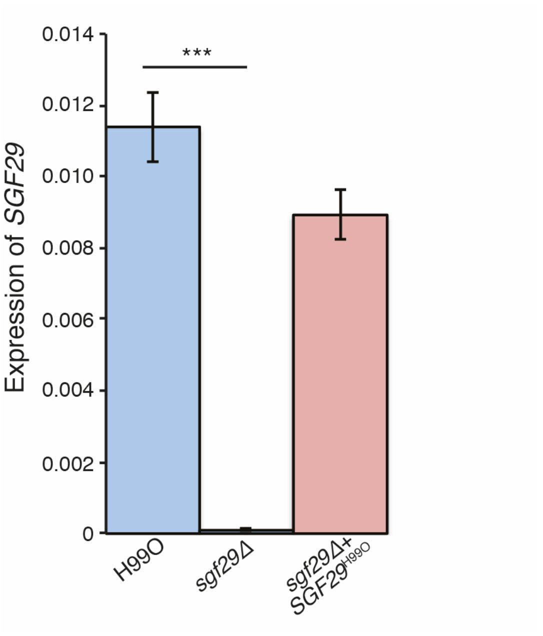

Supplementary Figure 3. Insertion of SGF29 at the Safe Haven site in a *sgf29*Δ strain has the same level of expression of *SGF29* compared with wild type. qRT-PCR indicated that expression of *SGF29* in the wildtype and complemented strain are not significantly different, while, as expected there is no expression of *SGF29* in the mutant strain. Values show mean, error bars show S.E.M, ***=P<0.001.

Supplementary Table 1: SNPs identified in this study

Identifier	Chr:Position	Mutation	Strains	Genes affected
**1**	**1**:825,487	G:A	H99O and derivatives	Intron of *CNAG_00321*, dRaptor
**2**	**2**:1,568,847	T:C	H99O and derivatives	*CNAG_04078*, Phe3Ser, oligosaccharyltransferase complex subunit gamma
**3**	**11**:241,670	A:G	H99O and derivatives	*CNAG_01541*, synonymous, plus 3' UTR of *CNAG_01542*
**4**	**13**:529,185	G:C	H99O and derivatives	*CNAG_06456*, Glu298Gln, hypothetical containing PPR repeat
**5**	**2**:868,724	G:A	H99L and derivatives	Intergenic, centromere
**6**	**1**:2,234,353	T:C	H99L and derivatives	3' UTR of *CNAG_00843*, salicylate hydroxylase
**7**	**3**:743,303	T:C	H99L and derivatives	Intron of *CNAG_12267* and *CNAG_02799* dihydroxyacetone kinase
**8**	**5**:1,525,128	A:G	H99L and derivatives	Intergenic, between *CNAG_00976* and *CNAG_00975*
**9**	**4**:1,059,881	C:G	H99L and derivatives	Intron of CNAG_05325, hypothetical, glycosyl hydrolase family 88
**10**	**5**:688,951	A:T	H99S	*CNAG_12447*, miscRNA
**11**	**10**:344,002	T:C	H99S	*CNAG_12900*, miscRNA
**12**	**11**:1,254,214	A:G	H99L$_{A}$	Intergenic
**13**	**1**:1,759,494	C:T	H99L$_{B}$	*CNAG_00677*, Arg82*, E3 ubiquitin ligase, Uhrf1
**14**	**13**:148,052	G:A	H99L$_{B}$	*CNAG_06308*, synonymous
**15**	**2**:1,036,539	A:G	H99L$_{C}$	Intron of *CNAG_03863*, protein arginine N-methyltransferase 1
**16**	**12**:313,138	T:G	H99L$_{C}$	3' UTR of *CNAG_06103*, RNA binding protein
**17**	**5**:608,311	A:C	H99L$_{D}$	*CNAG_01317*, Ser257Ala, hypothetical containing HLH domain
**18**	**7**:333,727	G:A	H99L$_{D}$	Intergenic, 2 bp away from 3' UTR of *CNAG_06633*
**19**	**12**:60,740	A:C	H99L$_{D}$	*CNAG_06006*, Tyr424*, hypothetical
**20**	**13**:400,284	A:T	H99L$_{D}$	5' UTR of *CNAG_06403*, hypothetical
**21**	**2**:84,393	T:C	H99L$_{E}$	*CNAG_06769*, Ser39Pro, DNA polymerase gamma 1
**22**	**1**:2,192,797	T:G	H99L$_{F}$	*CNAG_00826*, synonymous, plus 3' UTR of *CNAG_00825*, solute carrier family 25 (mitochondrial folate transporter), member 32
**23**	**14**:558,360	T:A	H99L$_{F}$	*CNAG_05527*, Ser490Thr, senataxin helicase
**24**	**1**:972,122	C:T	H99W lineage	Intergenic
**25**	**5**:3,681	C:T	H99W lineage	Intergenic
**26**	**5**:1,286,519	G:A	H99W lineage	*CNAG_01069*, synonymous
**27**	**11**:1,457,622	G:A	H99W lineage	Intron of *CNAG_01987*, carbohydrate binding protein
**28**	**10**:174,524	C:T	H99ED and derivatives	*CNAG_04864*, synonymous
**29**	**11**:223,290	G:T	H99ED and derivatives	*CNAG_01536*, synonymous
**30**	**11**:375,655	C:T	H99ED and derivatives	*CNAG_01594*, Thr64Ile, glycine dehydrogenase
**31**	**2**:195,745	G:A	H99C	*CNAG_06732*, synonymous
**32**	**2**:1,546,222	G:C	H99C	*CNAG_04070*, Leu849Val, exonuclease and 3' UTR of *CNAG_04069*, U4/U6 small nuclear ribonucleoprotein PRP31

Supplementary Table 2: INDELs identified in this study

Identifier	Chr:Position	Mutation	Strains	Genes affected
**A**	**2**:1,059,379	A:ACCTACATTCGTTACTG	H99O and derivatives	5’ UTR of *CNAG_03873*, hypothetical, potentially DNA binding
**B**	**6**:538,398	ACTC:A	H99O and derivatives	*CNAG_07634*, Glu1054_Leu1055 delete Glu, hypothetical
**C**	**8**:1,289,136	CG:C	H99O and derivatives	Intergenic
**D**	**14**:497,587	TA:T	H99S	Intron of *CNAG_05505*, serine/threonine-protein phosphatase 2A activator 2
**E**	**11**:550,343	CT:C	H99S	*CNAG_07595*, frameshift, hypothetical
**F**	**1**:1,859,705	C:CT	H99L$_A$	3' UTR of *CNAG_00711*, hypothetical
**G**	**1**:857,091	C:CAA	H99L$_C$	Intergenic
**H**	**1**:12,714	G:GA	H99W lineage	Intergenic
**I**	**2**:99,355	C:CCTGGCCG	H99W lineage	*CNAG_06765*, *LMP1,* frameshift
**J**	**2**:208,187	CGCAT:C	H99W lineage	3’ UTR of *CNAG_06730*, CMGC/GSK protein kinase
**K**	**7**:1,361,217	G:GA	H99W lineage	3’ UTR of *CNAG_05970*, STE/STE20/PAKA protein kinase
**L**	**9**:5,478	C:CA	H99W	Intergenic
**M**	**7**:150,814	C:CT	H99ED and derivatives	Intergenic
**N**	**7**:285,170	TCCACCACCACCACCA:T	H99E & H99C	3’ UTR of *CNAG_06614*, glycerophosphodiesterase
**O**	**1**:2,001,904	GT:G	H99C	Intron of *CNAG_00762*, diphthamide biosynthesis protein 1
**P**	**11**:372,742	AT:A	H99C	Intergenic, adjacent to 3' UTR of *CNAG_01592*, protein-S-isoprenylcysteine O-methyltransferase
SGF29	**13:** 367,683	734 bp deletion	H99L lineage	CNAG_06392, *SGF29*, 734 bp deletion

Supplementary Table 3: Fungal strains used in this study

Strain	Genotype
H99O	Wild-type
H99S	Wild-type
H99W	Wild-type
H99ED	Wild-type
H99E	Wild-type
H99C	Wild-type
H99L	Wild-type
H99L_A	Wild-type
H99L_B	Wild-type
H99L_C	Wild-type
H99L_D	Wild-type
H99L_E	Wild-type
H99L_F	Wild-type
ghh1Δ	*GHH1* deletion in H99O background
ghh1Δ+GHH1	*ghh1Δ* complemented with *GHH1* in Safe Haven
sgf29Δ	*SGF29* deletion in H99O background
sgf29Δ+SGF29^{H99O}	*sgf29Δ* complemented with H99O *SGF29* in Safe Haven
sgf29Δ+SGF29^{H99L}	*sgf29Δ* complemented with H99L *SGF29* in Safe Haven

Supplementary Table 4: Plasmids used in this study

Strain	Description
pJAF1	*NEO* resistance cassette vector
pSDMA25	*NAT* vector for targeted integration at Safe Haven
pKLO7	*GHH1* (H99O) in pSDMA25
pKLO10	*GHH1* (H99L) in pSDMA25
pKLO13	*SGF29* (H99O) in pSDMA25
pSRS6	*SGF29* (H99L) in pSDMA25

Supplementary Table 5: Primers used in this study

Primer	Name	Sequence
**UQ3372**	H99 family SNP1 fwd	GACTAGCGGATGGGACAGTG
**UQ3373**	H99 family SNP1 rev	GAGGATCTGATGGAGGCAGC
**UQ3374**	H99 family SNP2 fwd	CAAATGGGCACCACGAAAGG
**UQ3375**	H99 family SNP2 rev	ATGTACGCACTGGCATGGTT
**UQ3376**	H99 family SNP3 fwd	TTCTTCTTCCTCCCACACGC
**UQ3377**	H99 family SNP3 rev	CACCAATGTAACCTTGCGGC
**UQ3378**	H99 family SNP4 fwd	TTGTTCCCAACACTGCCACT
**UQ3379**	H99 family SNP4 rev	GAATTCTGGGATGGCGCTCT
**UQ3380**	H99 family SNP6 fwd	CAGGAGGCTAGAGATGCTGC
**UQ3381**	H99 family SNP6 rev	TGTTTGTTCAGCCAACCCCT
**UQ3382**	H99 family SNP7 fwd	GTGGGGCCTGGACTGTTATC
**UQ3383**	H99 family SNP7 rev	TGAGAATATGCGGACGAGGC
**UQ3384**	H99 family SNP8 fwd	AAAGCGACAACTGAGGTATGC
**UQ3385**	H99 family SNP8 rev	GTAGCACCTGTGAGGAAGACG
**UQ3386**	H99 family SNP9 fwd	CGAGGTGAGAGCCATATTTCG
**UQ3387**	H99 family SNP9 rev	GGTATGACGACGCTGAAAAGC
**UQ3388**	H99 family SNP10 fwd	CCCTCACATCATTCCCACTCG
**UQ3389**	H99 family SNP10 rev	CTCCTGGGTTTCTCCTGAAGC
**UQ3390**	H99 family SNP11 fwd	GTCACTTTCCGGTTGTCTTCC
**UQ3391**	H99 family SNP11 rev	CAAACGCTTTCGTCAAATCCC
**UQ3392**	H99 family SNP22 fwd	CACCGTCACCGACAATCTACC
**UQ3393**	H99 family SNP22 rev	TTTGCTCGTGAACAACATGGG
**UQ3394**	H99 family SNP23 fwd	CGGTGAAGAGAAAGGATCTGG
**UQ3395**	H99 family SNP23 rev	AAGGGTGCGAGAGATTTGAGC
**UQ3396**	H99 family SNP13 fwd	ACCTTCAGCTTCCAGTCTAGC
**UQ3397**	H99 family SNP13 rev	CACGGATTATGGACCCTGACG
**UQ3398**	H99 family SNP14 fwd	AGGTATGGGAAAGGAAATGCG
**UQ3399**	H99 family SNP14 rev	AAATCCGCCCACATGAACAGG
**UQ3400**	H99 family SNP17 fwd	TGGTGGGATCTGACATACACG
**UQ3401**	H99 family SNP17 rev	TGAGATTGCCCAACTCCTTCC
**UQ3402**	H99 family SNP18 fwd	TGTAGCTGAAAGGGGCAAAGC
**UQ3403**	H99 family SNP18 rev	AGCATGAGAGGAAACGTCTGG
**UQ3404**	H99 family SNP19 fwd	TATCGCAGAGACCATTGTTCC
**UQ3405**	H99 family SNP19 rev	GTCTTGTGCCATCCCAAATCC

**UQ3406**	H99 family SNP20 fwd	AGGTAGGAGGGTAGTGTTGGG
**UQ3407**	H99 family SNP20 rev	GTTTGAAGTGAGCGTGGAAGC
**UQ3408**	H99 family SNP21 fwd	ATATGCAACTGTCCCGATCCC
**UQ3409**	H99 family SNP21 rev	ATGCGGATGAGGTAACCTTGG
**UQ3410**	H99 family SNP12 fwd	TACCTCATGGAGCTGACTTCC
**UQ3411**	H99 family SNP12 rev	ATTGCCATCAACGAGTAGAGC
**UQ3412**	H99 family SNP15 fwd	GATGTTCCTGCTCCTCCTACG
**UQ3413**	H99 family SNP15 rev	TCCCAACTCCCGAAATAACCG
**UQ3414**	H99 family SNP16 fwd	AGCGGGAACTTTGTATCAACC
**UQ3415**	H99 family SNP16 rev	GCGGGAGATGGATGAAGATGG
**UQ3420**	H99 family SNP24 fwd	GGTCCATGCCATCGAAAACCC
**UQ3421**	H99 family SNP24 rev	TCTGTTGAGAGAAGGGTAGCG
**UQ3422**	H99 family SNP26 fwd	TGCGAGATGCTCAACAGATGG
**UQ3423**	H99 family SNP26 rev	TGTCTCCTCCAAGACTTCAGC
**UQ3424**	H99 family SNP27 fwd	GCTCTGGGAGGGTTATTGTCG
**UQ3425**	H99 family SNP27 rev	ATCCATCCCTGCTCCAACTCC
**UQ3426**	H99 family SNP28 fwd	AGCGGCGGAAATAGAAACACC
**UQ3427**	H99 family SNP28 rev	CTGAGTCGTCACAGGAGAAGG
**UQ3428**	H99 family SNP29 fwd	AGGCCAAAGTTGACGTAATCC
**UQ3429**	H99 family SNP29 rev	TCGATGATCTTTCGGAATGGC
**UQ3430**	H99 family SNP30 fwd	TTATCAGTGCCGATCCAGTGC
**UQ3431**	H99 family SNP30 rev	ATAAACGTCAGGCATGTCACG
**UQ3432**	H99 family SNP31 fwd	GATGCTGAGCGAATACAAGCC
**UQ3433**	H99 family SNP31 rev	TTCCAACCGAGCCAGAATACG
**UQ3434**	H99 family SNP32 fwd	AGCCAGTTTGTGATGCTTTGC
**UQ3435**	H99 family SNP32 rev	GGATGCGGATGAAGTGAATGC
**UQ3448**	H99 family Indel G fwd	CCGCTGGGTCAACCAAAATGG
**UQ3449**	H99 family Indel G rev	GAATCTGCACTGACCGATACC
**UQ3450**	H99 family Indel F fwd	ACCTCGACTGAGCTACATCCC
**UQ3451**	H99 family Indel F rev	TCACCATACCTGACCATACCG
**UQ3452**	H99 family Indel N fwd	CACTTTCGACATTCCCGTTCC
**UQ3453**	H99 family Indel N rev	GTCCAGTATCCAGCAGTAGCG
**UQ3454**	H99 family Indel O fwd	CAAAGCTGCGACCTCTAGACC
**UQ3455**	H99 family Indel O rev	AGAGATGAGGGGTGTCAATGC
**UQ3456**	H99 family Indel P fwd	GTTTCTTCTACTGGGCTGTGG
**UQ3457**	H99 family Indel P rev	GAGGATGAGGACAGTGACAGC
**UQ3474**	H99 family Indel L fwd	CTGGGTGGTAGTAGAGTTGCG
**UQ3475**	H99 family Indel L rev	GCCTCCCTGAAGTCCATTACG
**UQ3494**	*GHH1* 5' flank forward	TGACTACTCGAGGCACTTAGC

**UQ3219**	*GHH1*5' flank reverse	CGTGTTAATACAGATAAACCAGCTGGCGATGATGCGTGAGATG
**UQ3144**	*GHH1* 5' neo forward	ATCGCCAGCTGGTTTATCTGTATTAACACGGAAGAGATGTAG
**UQ3288**	*GHH1* 3' neo reverse	GACTGCAGATGAACCTGGGACGCTGCGAGGATGTGAGCTGGA
**UQ3289**	*GHH1* 3' flank forward	TCCAGCTCACATCCTCGCAGCGTCCCAGGTTCATCTGCAGTC
**UQ3483**	*GHH1* 3' flank reverse	TCACTAGAGGAACCGGCTTCG
**UQ3495**	*GHH1* 3' flank reverse (cloning)	TCATCCACTAGTTGCCTCAGC
**UQ3484**	*GHH1* sequencing primer 1	TGCTCCCACTCCCTCTTGATC
**UQ3485**	*GHH1* sequencing primer 2	TCATCGACAACCTCGTGAGAC
**UQ3486**	*GHH1* sequencing primer 3	ACCTCCAAGAATACACGGTCG
**UQ3148**	*GHH1* 5' diagnostic	AGCATGTGGAGACAGTTTGAG
**UQ3149**	*GHH1* 3' diagnostic	TTGAGCTGCTTGATGGCGATG
**UQ3574**	*SGF29* 5' flank forward	CTGTAGAGCTCCTCGAAATAC
**UQ2928**	*SGF29* 5' flank reverse	CCAGCTCACATCCTCGCAGCGTTGCCAAGTACGCTCCAGT
**UQ2929**	*SGF29* neo marker forward	ACTGGAGCGTACTTGGCAACGCTGCGAGGATGTGAGCTGG
**UQ2930**	*SGF29* neo marker reverse	GAATACAAATAGCCTATACCGGTTTATCTGTATTAACACG
**UQ2931**	*SGF29*' flank forward	CGTGTTAATACAGATAAACCGGTATAGGCTATTTGTATTC
**UQ3575**	*SGF29* 3' flank reverse	CCATACCTAGGCCATCCATAC
**UQ2933**	*SGF29* 5' diagnostic	TTTGATAGGGACGTACTTCTT
**UQ3482**	*SGF29* sequencing primer	CTCCTCGAAATACGACTGGTG
